# LRRK2 in Drosophila Melanogaster Model: Insights into Cellular Dysfunction and Neuroinflammation in Parkinson’s Disease

**DOI:** 10.3390/ijms26052093

**Published:** 2025-02-27

**Authors:** Cristina Ciampelli, Grazia Galleri, Manuela Galioto, Paolo Mereu, Monica Pirastru, Roberto Bernardoni, Diego Albani, Claudia Crosio, Ciro Iaccarino

**Affiliations:** 1 Department of Biomedical Sciences, University of Sassari, via Francesco Muroni 25, 07100 Sassari, Italy; c.ciampelli@phd.uniss.it (C.C.); pmereu@uniss.it (P.M.); pirastru@uniss.it (M.P.); ccrosio@uniss.it (C.C.); 2 Department Pharmacy and Biotechnology, University of Bologna, 40126 Bologna, Italy; roberto.bernardoni@unibo.it; 3 Department of Agricultural Sciences, University of Sassari, 07100 Sassari, Italy

**Keywords:** Parkinson’s disease, LRRK2, Drosophila, Attacin-A

## Abstract

Parkinson’s disease (PD) is a fatal neurodegenerative disease for which there are no still effective treatments able to stop or slow down neurodegeneration. To date, pathological mutations in the leucine-rich repeat kinase 2 (LRRK2) gene have been identified as the major genetic cause of PD, although the molecular mechanism responsible for the loss of dopaminergic neurons is still cryptic. In this review, we explore the contribution of Drosophila models to the elucidation of LRRK2 function in different cellular pathways in either neurons or glial cells. Importantly, recent studies have shown that LRRK2 is highly expressed in immunocompetent cells, including astrocytes and microglia in the brain, compared to neuronal expression. LRRK2 mutations are also strongly associated with the development of inflammatory diseases and the production of inflammatory molecules. Using Drosophila models, this paper shows that a genetic reduction of the inflammatory response protects flies from the neurodegeneration induced by LRRK2 pathological mutant expression.

## 1. Introduction

Parkinson’s disease (PD) is the second most common neurological disease and the most common movement disorder worldwide. PD clinical manifestations include resting tremor, muscle rigidity, bradykinesia, and postural instability. Aging remains the biggest risk factor for developing idiopathic PD and in fact, the global burden of PD is increasing with socioeconomic progress and the aging of the world’s population.

The main pathological sign of PD is the loss of dopaminergic neurons in the Substantia Nigra pars compacta (SNpc) that project to the dorsal putamen of the striatum, resulting in the progressive impairment of voluntary motor control. Another neuropathological feature of PD is the accumulation in neurons of cytoplasmic inclusions called Lewy bodies (LB), mainly composed of misfolded α-synuclein [1]. This was also the first gene mutation associated with familial PD [1]. Importantly, no current treatments/therapies are able to stop or delay dopaminergic neuronal degeneration in PD patients, only relieve motor symptoms. PD is generally believed to be the result of a combination of age, genetics, and environmental factors. Currently, monogenic PD accounts for approximately 5% of total cases while the other 95% fall into the category of idiopathic PD. In familial PD cases, pathological mutations in the leucine-rich repeat kinase 2 (LRRK2) gene are the most common genetic cause, reaching up to 40% in some ethnic groups, such as Ashkenazi Jewish and North African Arab Berbers and, in addition, LRRK2 is also found mutated in approximately 1–3% of idiopathic PD cases [2,3]. LRRK2 pathological mutations are autosomal dominant and, importantly, PD patients carrying LRRK2 mutations are clinically and neuropathologically indistinguishable from idiopathic patients, although they exhibit a prevalence of tau aggregates [4], supporting the idea that the elucidation of the physiological and pathological role of LRRK2 may strongly contribute to the discovery of the molecular mechanisms of neurodegeneration in PD, possibly leading to new therapeutical approaches.

**Figure 1:**
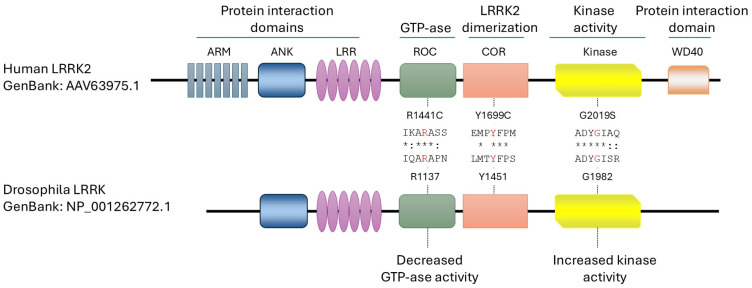
Schematic representation of the functional domains of human LRRK2 (hLRRK2) compared to Drosophila LRRK (dLRRK). The key functional domains are highly conserved between human and Drosophila, including the aminoacidic residues mutated in the human pathological variants (R114C, Y1669C, and G2019S). Armadillo (ARM), Ankyrin (ANK), Leucine-rich-repeat (LRR), Ras of complex proteins (ROC), C-terminal of ROC (COR), Kinase, and WD40 domains.

The LRRK2 gene, located on chromosome 12 (12q12 area) and composed of 51 exons, encodes a large protein of 288kDa belonging to the Roco family. Roco proteins are characterized by the presence of kinase domain associated with an ROC (Ras of complex proteins) domain and a COR (C-terminal of ROC) domain [5] (Figure 1). Moreover, LRRK2 is a multifunctional protein containing additional domains, including armadillo, ankyrin, and leucine-rich repeat motifs in the N-terminus and the WD40 domain in the C-terminus of the protein (Figure 1). These domains are likely involved in the interaction with different proteins and regulate the physiological function of LRRK2 [6].

The phosphorylation activity of the LRRK2 protein is considered a key feature of its function. LRRK2 is a serine-threonine kinase capable of both autophosphorylation at its serine-1292 residue and phosphorylation of target substrates, including Rab GTPases [7]. In addition, ROC and COR domains modulate the kinase domain controlling protein dimerization, kinase activity, and interactions with other regulatory proteins [8].

LRRK2 gene mutations were first discovered to be associated with PD in 2004 [9,10]. The most prevalent mutation is G2019S, which is highly prevalent in North African and Middle Eastern ethnic groups, including Ashkenazi Jews and Arabs (27). Interestingly, all pathological mutations identified to date are located in the two catalytic domains of the proteins and increase the kinase activity: G2019S and I2020T are located in the kinase domain, R1441C/G/H are in the ROC domain, and Y1699C is in the COR domain [6]. LRRK2 is expressed in the brain but also in peripheral organs including the liver, kidneys, heart, and lungs, with different levels of expression in different cell types. In the mammalian brain, LRRK2 mRNA and protein are highly expressed in brain areas innervated by dopamine neurons; in fact, compared to other PD-associated genes, there is very low LRRK2 mRNA and protein level in the dopamine neurons themselves [11]. In addition, LRRK2 expression is greater in microglia and astrocytes compared to neurons, suggesting a possible non-cell autonomous mechanism of toxicity [11].

LRRK2 protein (with its multiple enzymatic activities and domains with different functions) has been implicated in many different biological processes, although most experimental results converge on a critical role in the control of vesicle trafficking [12]. In fact, a sub-set of Rab proteins are considered the most likely substrates of LRRK2 kinase activity [13]. Steger and colleagues were the first to demonstrate that LRRK2 phosphorylates a subset of Rab GTPases on a conserved residue in their switch-II domains [14]. About 70 Rab GTPase family members are expressed in humans and are considered critical regulators of intracellular vesicle trafficking. Most of the Rabs are ubiquitously expressed proteins and have been implicated in vesicle formation, vesicle motility/delivery along cytoskeleton elements, and finally vesicle docking/fusion at target membranes through the recruitment of several effectors that may positively or negatively regulate the Rab function [13].

Neurobiological alterations associated with PD pathogenesis focus on altered protein balance, mitochondrial dysfunction, oxidative stress, and, not least, neuroinflammation [11]. In fact, neuroinflammation is one of the common features of several neurodegenerative diseases, including PD. Interestingly, according to high LRRK2 expression in immune competent cells, genetic variations in the LRRK2 gene have been implicated in susceptibility to infectious agents such as Mycobacterium Leprae [15] and Mycobacterium Tuberculosis [16] and associated with inflammatory diseases including Systemic Lupus Erythematosus [17] and Inflammatory Bowel Disorders (IBD) [18]; patients with IBD have a higher increased risk of PD.

Importantly, in the brain, the expression of pathological LRRK2 mutations leads to an increase in the production of inflammatory cytokines and chemokines in response to different stimuli, both in microglia and astrocytes [19,20,21,22]. In contrast, LRRK2 knock-out [23,24] or pharmacological LRRK2 inhibition [23,25,26,27] determine a reduction of inflammatory mediators, further supporting the critical role of LRRK2 in the control of inflammation in general and, in particular, in the brain. Interestingly, inflammation has been extensively implicated in the development of PD [28] and other parkinsonisms [29]. Indeed, reactive microglia and activated complement components are evident in affected brain regions of PD patients and this correlates with several markers of chronic inflammation [28]. In addition to inflammatory molecules, other neuromodulators secreted by non-dopaminergic cells may affect the physiology or survival of dopaminergic neurons [30]. For example, the neuropeptide orexin, produced by orexigenic neurons in the lateral parts of the hypothalamus, is involved in the modulation of motor control and has a neuroprotective effect on the dopaminergic neurons highlighted in preclinical models of PD [31].

Based on the previous considerations, the physiological and pathological function of LRRK2 is still cryptic. Unfortunately, LRRK2 transgenic rodent models have not contributed significantly to a clear definition of LRRK2 function, as most transgenic rodents, collectively, do not show overt nigrostriatal neurodegeneration or consistent alterations in the physiology of dopaminergic neurons [32]. Rather, LRRK2 rodent models appear to be a useful tool for testing the dual-hit or multiple-hit hypothesis of PD. Non-rodent models have been widely used to study LRRK2 function and disease mechanism [33]. The expression of human LRRK2 variants in non-rodent organisms has made it possible to directly observe the effects of mutations on behavior, neurodegeneration, and alteration of specific cellular pathways. For example, zebrafish, Drosophila, or *C. elegans* expressing mutated forms of LRRK2 often show reductions in longevity, alterations in movement, and neuronal degeneration similar to those observed in patients with PD [33]. A particular advantage of using simpler organisms as a model is the possibility of conducting large-scale genetic screenings to evaluate gene pathway interaction and easily test possible therapeutic options. These approaches can identify other genes or chemical compounds that modify the effects of LRRK2 mutations, providing potential targets for new therapeutic treatments. Furthermore, with the advanced imaging techniques and genetic tools available in simpler models, it is possible to examine in detail the cellular and molecular changes induced by LRRK2 mutations. For various reasons, the role of LRRK2 in neurodegeneration in non-rodent models has been mainly analyzed using Drosophila models, with few research articles on other in vivo models, such as zebrafish and *C. elegans*. Moreover, we include original experimental data suggesting an important contribution of inflammatory molecules in the neurodegeneration in LRRK2 Drosophila models.

## 2. Drosophila LRRK2 Models

Compared with higher eukaryote model organisms, such as rodents, the *Drosophila melanogaster* model offers some advantages to investigate brain diseases: low-cost maintenance, fast reproduction, and short experimental cycle, large availability of genetic tools for gene manipulation and gene interaction studies [34,35]. Obviously, Drosophila and higher eukaryotes differ in terms of neuronal structures, organization, ratio between neuronal, and glial cells, making the higher eukaryotes a more appropriate model to investigate the physiology of dopaminergic neurons and to translate the experimental findings to human patients. However, between Drosophila and humans, a large number of genes have high levels of conservation, meaning that both species share common gene functions/pathways. Recently, both the whole-brain connectome of an adult fly, containing over 130,000 neurons and millions of synaptic connections [36,37] and network statistics of the whole-brain connectome have become available [38], opening new important scenarios to brain studies. In Drosophila, a sole LRRK ortholog (CG5483) has been identified by genome sequence analysis, designated as dLRRK, while in human and high eukaryotes, two members of the leucine-rich repeat kinase family exist (LRRK1 and LRRK2). Drosophilia dLRRK shares 24% identity and 38% similarity to the human aminoacidic sequence. Obviously, the kinase domain shows a higher identity and similarity between dLRRK and hLRRK2, and importantly, analogous critical amino acids for LRRK2 kinase function, including proton acceptor (D1994) and ATP binding site (K1906) and the most common pathological mutations (R1441C, Y1699C, and G2019S) are highly conserved [39] (Figure 1). Similarly to human expression data, dLRRK is largely ubiquitously expressed in both the head and body tissues of Drosophila [40].

### 2.1. Loss of Function Mutants

Different dLRRK deletion mutants have been generated (summarised in Table 1). In 2007, Lee et al. [40] first showed that the dLRRK loss-of-function mutants exhibit a severely impaired locomotor activity as early as 3 days after eclosion and becoming more severe with age. Moreover, this locomotor phenotype is associated with a significant reduction in tyrosine hydroxylase immunostaining and shrunken morphology of DA neurons [40]. However, another study by Imai et al. [41] in 2008 showed no significant change in TH-positive neurons in dLRRK (−/−) flies, although an increase in DA content was observed, likely due to changes of dopamine transmission, storage, or metabolism. Those dLRRK (−/−) animals are viable, despite the females having reduced fertility. Moreover, KO animals appear to be more resistant to oxidative stress inducers such as paraquat or H_2_O_2_ [41]. A dispensable role in terms of life span and survival of dopaminergic neurons was confirmed by other independent groups using different Drosophila mutants [39,42], while the role of dLRRK in the control of oxidative stress is still debated. For instance, a truncated dLRRK variant lacking C-terminal kinase appear more sensitive to H_2_O_2_-induced stress but not to paraquat, rotenone, and beta-mercaptoethanol [39]. It is possible that the different genetic backgrounds, large deletion in dLRR genomic regions that may affect the expression of neighboring genes or different nutrient conditions, may account for the divergent results related to oxidative stress.

### 2.2. Gain of Function Mutants

In human, LRRK2 pathological mutants are autosomal dominant, strongly suggesting a LRRK2 gain of function. In flies, different LRRK2 isoforms have been expressed to model human disease phenotype under the control of different cell-type specific GAL4 drivers (Table 1). In general, results from several laboratories agree that when the LRRK2 isoforms are expressed in dopaminergic neurons, either by Tyrosine Hydroxylase (TH)- or DOPA decarboxylase (Ddc)-GAL4 drivers, a significant pathological phenotype, including climbing defects and a decrease in dopaminergic neuron number, is detected. Liu et al. first showed that the expression of either LRRK2 WT or LRRK2-G2019S in neurons resulted in a selective loss of dopaminergic neurons paralleled by locomotor dysfunction and early mortality. Expression of the G2019S mutant caused a more severe parkinsonism-like phenotype compared to equivalent expression levels of wild-type LRRK2. Treatment with L-DOPA improved mutant LRRK2-induced locomotor dysfunction but was ineffective in preventing the loss of TH-positive neurons [43]. The late-onset loss of dopaminergic (DA) neurons in selected clusters accompanied by locomotion deficits has been confirmed by several independent groups [44,45,46]. Interestingly, this phenotype is also observed when expressing dLRRK isoforms mutated in the same critical amino acids [41] and specific Rab phosphorylation is detected in Drosophila over-expressing LRRK2 isoforms comparable to high eukaryotic systems, further validating the fly models [50,51].

More controversial is the toxicity in flies when LRRK2 is driven by pan-neuronal expression ranging from a less severe phonotype compared to specific expression in DA neurons [43] to an absence of a significant phenotype [48]. This paradox may be easily explained by the lower expression of LRRK2 proteins in DA neurons under the control of a pan-neuronal-GAL4, such as the elav-GAL4 driver. In our case, we could detect a strong and consistent pathological phenotype using ubiquitous promoter either tubulin or actin. This could be particularly relevant considering that, both in human and Drosophila, LRRK is ubiquitously expressed and, importantly, mammalian studies have shown that LRRK2 expression occurs at lower level in dopaminergic neurons compared to other brain areas. In addition, in human LRRK2 is highly enriched in immune competent cells, including microglia and astrocytes, strongly suggesting a possible non-cell autonomous mechanism of neuronal toxicity. Interestingly, in our Drosophila model, the ubiquitous expression of LRRK2 leads to a strong increase of inflammatory signals that may partially mediate the neuronal toxicity [48]. Moreover, in Drosophila, pan-glial LRRK2 expression by the repo-GAL4 driver leads to both inflammation and locomotor defects that parallels neurodegeneration [48]; endogenous dLRRK depletion by dLRRK-RNAi under the control of the repo-GAL4 driver causes locomotion deficits, glial apoptosis, and DA neurodegeneration in an age-dependent manner [49]. Furthermore, neuron-glial bone morphogenic protein (BMP) signaling cascade is critical for mediating age-dependent neurodegeneration in two different LRRK2 Drosophila models [47].

Taken together, these findings demonstrate a functional role of hLRRK2/dLRRK in Drosophila glial cells, suggesting a possible contribution to neurodegeneration.

## 3. Cellular Dysfunction in LRRK2 Drosophila Models

### 3.1. LRRK2 and Vesicle Trafficking

To date, the physiological and pathological function of LRRK2 is still cryptic, although most of experimental results converge on the critical role of LRRK2 in the regulation of vesicle trafficking [12]. In this regard, experimental Drosophila models recapitulate many of the alterations in vesicular trafficking that have been observed in higher eukaryotes (Table 2). For instance, in flies expressing the R1441C LRRK2 mutant, many synaptic vesicle proteins are differentially expressed, including synaptotagmin-1, syntaxin-1A, and RAB3 [52]. In addition, a phosphoproteome approach reveals an enhanced phosphorylation of several SV proteins, including synaptojanin-1 [52]. Finally, a protein-protein interaction screen in the fly brain confirms that LRRK2 robustly interacts with numerous SV proteins, including synaptojanin-1 and EndophilinA (EndoA) [52]. In high eukaryotes, LRRK2 directly phosphorylates synaptojanin1 in vitro, resulting in the disruption of endophilin-synaptojanin1 interaction and leading to a deregulation of SV trafficking in ventral midbrain neurons [53]. Furthermore, LRRK2 phosphorylates the EndoA BAR domain both in flies and eukaryotic cells; this phosphorylation impairs synaptic vesicle endocytosis [54]. Moreover, EndoA phosphoswitch mediated by LRRK2 seems critical for macroautophagy at presynaptic terminals [54]. The postsynaptic overexpression of either the fly or human LRRK2 transgene induces a retrograde enhancement of presynaptic neurotransmitter release by increasing the vesicle size of the readily releasable pool, while the postsynaptic knockdown of the dLRRK shows an opposite effect [55]. LRRK2 modulates the phosphorylation of the μ2 subunit of the adaptor protein AP2 (AP2M1), a core component of the clathrin-mediated endocytic machinery both in human SH-SY5Y cells and mouse neurons and tissues [56]. Moreover, in Drosophila, the LRRK2-dependent phosphorylation of AP2M1 mediates dopaminergic neurodegeneration [56]. Similarly, in mouse models or high eukaryotic cells, the expression of LRRK2 modifies the recruitment and phosphorylation of AP2. Furthermore, the phosphorylation of AP2 mediated by LRRK2 R1441C pathogenic mutation results in an impaired clathrin-mediated endocytosis [57].

Recently, experiments in high eukaryotic models demonstrate that the VPS35 pathological mutant (D620N) enhances the LRRK2 kinase activity towards the phosphorylation of different Rabs under basal conditions, strongly suggesting that LRRK2 and VP35 share common pathological pathways [58,59,60]. The genetic interaction between LRRK2 and VPS35 has been first demonstrated in Drosophila models. In fact, Linhart et al. in 2014 demonstrated that overexpression of VPS35 or VPS26 significantly protects against the LRRK2 pathological phenotype, both in terms of locomotion deficits and shortened lifespan, when expressed by Ddc-GAL4 drivers [61]. Similar results were obtained by analyzing the LRRK2 pathological phenotype in the eyes when the transgenes are both expressed by Glass Multiple Reporter (GMR)-GAL4 drivers [61]. Mechanistically, experimental evidence in Drosophila indicates that LRRK2 and VPS35 proteins cooperate in synaptic vesicle trafficking endocytosis through the regulation of the endosomal pathway [62].

Importantly, vesicle trafficking plays a prominent role in the regulation of autophagic processes and autophagic alterations likely lead to synaptic dysfunction and neurodegeneration [63]. Lysosome generation or fusion and lysosomal protein trafficking relay in vesicle dynamics. In Drosophila, dLRRK physically interacts with RAB7 and inhibits the RAB7-dependent perinuclear lysosome clustering [64]. On the contrary, the dLRRK G1914S mutant (analogous to LRRK2 G2019S) promotes lysosome clustering, suggesting an endo-lysosomal dysfunction in the LRRK2 mutant pathogenesis [64]. In Drosophila and high eukaryotic experimental models, the LRRK2-dependent EndoA phosphorylation plays a critical role in autophagy [54]. Mechanistically, phosphorylated EndoA creates curved membrane-protein docking sites that are capable of recruiting Atg3 [54]. Moreover, Sergio Hernandez-Diaz et al. have demonstrated, using Drosophila models, that also Endophilin-B (EndoB) is required for autophagosome biogenesis at the synapse, that EndoB functions downstream of EndoA and, finally, that the loss of EndoB blocks the autophagy induction promoted by the PD mutation LRRK2 G2019S [65]. Interestingly, LRRK2 G2019S-induced neurodegeneration in Drosophila is highly dependent on dietary amino acid content, since it can be alleviated by both lower and higher amino acid diets [66]. The protective effect of a moderately high amino acid diet in dopamine neuron loss and motor deficits seems to involve the stress-responsive activation of 5′-AMP-activated protein kinase (AMPK) and consequently neuroprotection induction of autophagy [66].

Different experimental evidence underlines a significant LRRK2 localization at Endoplasmic Reticulum (ER) exit sites [67]. ER exit sites are key hubs in the regulation of many physiological processes and organelle biogenesis, including lysosomes and mitochondria. For instance, in high eukaryotes, the endoplasmic reticulum contributes to lysosomal tubulation/sorting driven by LRRK2 pathological mutants [68] and, in Drosophila, altered ER-mitochondria contact impacts mitochondria calcium homeostasis and contributes to neurodegeneration in LRRK2 models [69].

A possible explanation of the complex phenotype associated with LRRK2 pathological mutant expression is that any alteration in vesicle trafficking may modulate several neuronal and not neuronal structures/functions. These range from the addition of membranes and proteins during neurite outgrowth or at mature synapses, to the release of inflammatory proteins by immune cells or neurotransmitters by differentiated neurons, and, not least, organelle biogenesis and function, such as lysosomes and mitochondria.

**Table 2:** LRRK2 and Vesicle Trafficking in Drosophila models.

Pathway/Mechanism	Model System	Effect/Outcome	Refs.
Synaptic protein alteration	Transgenic expression in DA neurons: UAS LRRK2 R1441C/Ddc-GAL4	Altered expression of synaptotagmin-1, syntaxin-1A, RAB3; enhanced phosphorylation of different SV proteins; LRRK2 interacts with synaptojanin-1 and EndoA.	[52]
Endocytosis	Lrrk loss-of-function mutants: (Lrrk^P1^[e03680] and Lrrk^EX2^). Ubiquity transgenic expression: UAS-LRRK2 WT or mutant/Da-GAL4	LRRK2 phosphorylates EndoA at BAR domain; impairs synaptic vesicle endocytosis; affects macroautophagy at synapses.	[54]
Synaptic Function	Lrrk loss-of-function mutants: (Lrrk^P1^[e03680]) or UAS-dLRRK-RNAi in different tissue. Transgenic expression in motor neurons or muscle of UAS-LRRK2 WT or mutants.	LRRK2 expression increased vesicle size of the readily releasable pool. LRRK2 knockdown shows opposite effect	[55]
Clathrin-mediated Endocytosis	Lrrk loss-of-function mutants: Lrrk^P1^[e03680] and Lrrk^EX2^	dLRRK KO leads to impairments in clathrin-mediated synaptic vesicle endocytosis and neurotransmission	[54]
VPS35 Interaction	Transgenic expression in dopaminergic neurons or eyes: UAS-LRRK2 mut or UAS-dVPS35-VPS26 lines/Ddc-GAL4 or GMR-GAL4. dLRRK2^−/−^ flies crossed with different dVPS35^+/−^ and dVPS35^−/−^ genetic backgrounds or expressing UAS-dVPS35 WT or mutant in different tissues.	VPS35 expression protects from LRRK2 pathology: improves locomotor deficits and lifespan. LRRK2 and VPS35 cooperate in endosomal pathway.	[61,62]
Lysosomal Function	Lrrk loss-of-function mutants: Lrrk^P1^[e03680]. Transgenic expression in follicle cells: UAS-dLRRK G1914S/CY2-GAL4	LRRK2 interacts with RAB7 and affects lysosome clustering	[64]
Autophagy	Lrrk loss-of-function mutants: Lrrk^P1^[e03680] and Lrrk^EX2^. Ubiquity transgenic expression: UAS-LRRK2 WT or mutant/Da-GAL4 Lrrk loss-of-function mutants: Lrrk^P1^[e03680] mutant. Transgenic expression in motor neurons or eyes: UAS-hLRRK2-G2019S/D42-GAL4 or GMR-GAL4	EndoA phosphorylation by LRRK2 is crucial for autophagy EndoB is required for autophagosome formation Loss of EndoB blocks G2019S-induced autophagy	[54,65]
ER Function	Transgenic expression in DA neurons: UAS-hLRRK2-G2019S/TH-GAL4	LRRK2 affects ER-mitochondria contact. LRRK2 impacts calcium homeostasis	[69]

### 3.2. LRRK2 and Protein Translation

The first evidence that LRRK2 might be implicated in the regulation of protein translation was provided by Imai et al. in 2008 using a Drosophila model [41]. Both dLRRK and hLRRK2 modulate the phosphorylation of the eukaryotic initiation factor 4E (eIF4E)-binding protein (4E-BP), thereby increasing the protein translation both in vivo and in vitro [41] (Table 3). By combining Drosophila and human neuron PD models, Martin et al. were able to demonstrate that LRRK2 could directly phosphorylate the ribosomal protein s15. Pathological LRRK2 mutants stimulate both cap-dependent and cap-independent mRNA translation, leading to a bulk increase in protein synthesis in Drosophila, which can be prevented by phosphodeficient T136A ribosomal protein s15 mutant [70].

As previously mentioned, neurodegeneration driven by the LRRK2 G2019S mutation in Drosophila is critically modulated by dietary amino acid content, with both amino acid restriction and supplementation effectively attenuating its progression [66]. The protective effect of a low amino acid diet on the LRRK2 pathological mutant phenotype seems to involve a significant reduction in aberrant protein synthesis, both in Drosophila and rat primary neurons [66]. The LRRK2 effect on cap-dependent translation in vitro and in vivo was confirmed by another study focused on the ability of LRRK2 to impact the translation of specific mRNAs that contain highly complex 5′-UTRs. In particular, the authors identified Furin 1 as one of the main LRRK2 translational target, which is specifically required to modulate the synaptic function of LRRK2 [55].

The results obtained in Drosophila were afterwards confirmed in human dopaminergic neurons differentiated from patient-derived induced pluripotent stem cells (iPSCs) where the altered translation due to G2019S LRRK2 expression leads to an increased expression of genes responsible for calcium influx [71]. In agreement with this result, an increase or decrease in mRNA translation was observed in G2019S LRRK2 and LRRK2 knock-out mouse brains, respectively [72]. Another group identified S6 kinase as an important gene modifier of LRRK2-induced neurotoxicity [73]. Phosphorylation of S6 stimulates protein synthesis at the ribosome, and the RNAi for S6 kinase significantly ameliorates the LRRK2 G2019S pathogenic phenotype [73]. Of course, the results should be treated with caution, since S6 kinase is able to phosphorylate several important substrates other than ribosomal proteins. Finally, an RNA-sequencing using mRNA isolated from dopaminergic neurons of different LRRK2 Drosophila transgenic animals identified genes involved in actin cytoskeleton reorganization and elongation of protein translation [74].

**Table 3:** LRRK2 and Protein Translation in Drosophila models.

Process/Pathway	Model System	Effect/Outcome	Refs.
LRRK2 phosphorylates 4E-BP	Transgenic expression in neurons or only in DA neurons: UAS-dLRRK WT or Y1383C or I1915T/Elav-GAL4 or Ddc-GAL4	Increased protein translation	[41]
LRRK2 phosphorylates ribosomal protein s15	Lrrk loss-of-function mutants: Lrrk^P1^[e03680] Transgenic expression in DA neurons UAS-hLRRK2 WT or mutants/Ddc-GAL4	Increased cap-dependent and cap-independent translation Bulk increase in protein synthesis	[70]
G2019S mutation & amino acid diet	Transgenic expression in neurons or only in DA neurons UAS-hLRRK2-G2019S/Ddc-GAL4 or elav-GAL4	Both amino acid restriction and supplementation attenuate LRRK2 neurodegeneration. Low amino acid diet reduces aberrant LRRK2-dependent protein synthesis.	[66]
LRRK2 impacts the translation of specific mRNAs	Lrrk loss-of-function mutants: Lrrk^P1^[e03680] Transgenic expression in muscle UAS hLRRK2/G14-GAL4	Affects translation of mRNAs with complex 5′-UTRs. Furin 1 was identified as main translational target.	[55]
S6 kinase as gene modifier	Transgenic UAS-hLRRK2-G2019S in dopaminergic neurons or muscle (Ddc-Gal4 or 24B-GAL4)	RNAi for S6 kinase ameliorates G2019S phenotype	[73]

### 3.3. LRRK2 Pathology and Therapeutic Drugs

LRRK2 pathogenic mutations are autosomal dominant and determine an increase in LRRK2 kinase activity. Consequently, the development and use of LRRK2 kinase inhibitors represent a primary strategy for therapeutic intervention (Table 4). To date, highly specific LRRK2 kinase inhibitors have been developed. These are able to cross the blood-brain barrier and to significantly reduce the LRRK2 kinase activity [75]. The major problem seems related to the side effects of these inhibitors, mainly in pneumocytes, likely due to the LRRK2 ubiquitous expression in the different tissues, including the lungs [75]. LRRK2 kinase inhibitors have been tested also in Drosophila models. Collectively, these experiments demonstrate that LRRK2 kinase inhibitors protect LRRK2 Drosophila models against LRRK2 neurotoxicity in terms of locomotion deficits and decrease in the number of dopaminergic neurons [76,77,78].

Interestingly, the LRRK2 kinase inhibitor PF-06447475 can also bind with high affinity to dLRRK and protects Drosophila against paraquat-induced locomotor impairment, life span reduction, and oxidative stress [79]. Further validating these results, PF-06447475 does not show any protective effect on phenotypes due to dLRRK RNAi [79]. Interestingly, parallel to the human condition, L-DOPA treatment in Drosophila improves LRRK2-induced locomotor impairment without affecting the decrease in TH-positive neurons [43].

Drosophila is a very convenient organism for a rapid evaluation of specific neuroprotective compounds or for a large chemical or genetic screening of potential neuroprotective agents. For instance, screening of a 640 compound FDA-approved drug library that could suppress neurite degeneration in LRRK2-G2019S Drosophila models led to the identification of Lovastatin as a compound able to restore motor disability and dopaminergic neuron loss in aged LRRK2-G2019S flies [44]. Mechanistically, Lovastatin acts by regulating the Akt/NRF2 pathway and, in particular, inhibits the downstream GSK3β activity by increasing the GSK3β (Ser9) phosphorylation [44].

The neuroprotective effects of compounds capable of reducing LRRK2 mRNA levels have been evaluated using Drosophila models. The AICAr compound regulates LRRK2 levels through ARE-mediated mRNA decay modulated by AUF1 binding and importantly AICAr treatment suppresses LRRK2 expression and rescues LRRK2-induced dopaminergic neurodegeneration and neuroinflammation in PD Drosophila and mouse models [80]. AICAr is the substrate of the protein encoded by ATIC gene and, in line with the previous findings, ATIC over-expression (that leads to a AICAr decrease) or KO (that leads to an AICAr increase) can reduce or increase the LRRK2 mRNA levels, respectively, in various experimental models, including yeast [80]. In the attempt to block LRRK2, inhibitors not targeting the LRRK2 kinase domain have also been developed or identified [81]. For instance, 5′-deoxyadenosylcobalamin (AdoCbl), a physiological form of the essential micronutrient vitamin B12, directly binds LRRK2, leading to the alterations of protein conformation, ATP binding, and disrupting LRRK2 dimerization [82]. Treatment with AdoCbl inhibits LRRK2 kinase activity in cultured cells and brain tissues, and, importantly, has a neuroprotective effect in cultured primary rodent neurons as well as in in vivo LRRK2 models, including Drosophila [82].

In Ddc-GAL4/LRRK2-G2019S-expressing flies, treatment with epigallocatechin gallate (EGCG), a green tea-derived catechin, ameliorates the loss of DA neurons and mitochondrial dysfunction due to LRRK2 G2019S expression [83]. Interestingly, the EGCG neuroprotective effect was abolished when AMP-activated protein kinase (AMPK) was genetically inactivated, suggesting that EGCG-mediated neuroprotection requires AMPK. Consistent with this finding, the EGCG protective effect was mimicked by pharmacological or genetic activation of AMPK [83]. Finally, we demonstrated that the Levetiracetam, a compound largely used in human epilepsy treatment, can significantly rescue the LRRK2 R1441C pathological effect in eucaryotic cells and Drosophila [48].

**Table 4:** LRRK2 pathology and therapeutic approaches tested in Drosophila models.

Compound	Therapeutic Target	Model System	Effects	Refs.
LRRK2 kinase inhibitors	LRRK2 pathway	dLRRK RNAi in DA neurons UAS-Lrrk-RNAi/TH-GAL4 Different LRRK2 transgenes: UAS-LRRK2 isoforms/TH or Ddc-GAL4	Reduces loss of dopaminergic neurons. Protects against paraquat toxic effects. Improves locomotor impairment. Reduces oxidative stress.	[76,77,78,79]
Lovastatin (FDA-approved drug as oral antilipemic agent)	Increases the GSK3β (Ser9) phosphorylation modulating the Akt/Nrf signalling pathway	Transgenic expression in DA neurons: UAS-LRRK2-G2019S/Ddc-GAL4	Restores motor disability. Prevents dopaminergic neuron loss.	[44]
AICAr	ARE-mediated mRNA decay	Transgenic expression in DA neurons: UAS-LRRK2-G2019S/Ddc-GAL4	Suppresses LRRK2 expression. Rescues neurodegeneration. Reduces neuroinflammation.	[79]
AdoCbl	LRRK2	Transgenic expression in DA neurons UAS-LRRK2-G2019S/TH-GAL4 Lrrk loss-of-function mutants Lrrk^P1^[e03680]	Binds directly LRRK2. Disrupts LRRK2 dimerization. Shows neuroprotective effects.	[82]
EGCG (Green tea derivative)	AMP-activated protein kinase (AMPK)	Transgenic expression in neurons, DA neurons or muscle: UAS-hLRRK2-G2019S/Elav-GAL4, ddc-GAL4 or 24B-GAL4	Ameliorates DA neuron loss. Improves mitochondrial function.	[83]
Levetiracetam (FDA-approved antiseizure drug)	SV2A and other neuronal targets	Ubiquity transgenic expression: UAS-LRRK2 R1441C/Actin-GAL4	Ameliorates DA neuron loss. Improves motor functions.	[48]

## 4. Is the PD LRRK2-Related Disease a Cell Autonomous Disease?

As discussed in the introduction, LRRK2 is expressed at low levels in dopaminergic neurons compared to other neuronal districts and is expressed at high levels in cells of the immune system, including microglia and astrocytes. Furthermore, the involvement of LRRK2 in some autoimmune diseases strongly suggests a prominent role of LRRK2 in inflammation and probably neuroinflammation. Growing evidence supports the idea that pathological interplay between peripheral and CNS innate immunity significantly contributes to the LRRK2 dependent neurotoxicity [84]. In the CNS, LRRK2 directly modules microglia/astrocyte physiological function while at peripheral level could, for instance, affect the gut-brain axis.

Compared to high eukaryotes, Drosophila show a simpler innate immune response that involves both humoral and cell-mediated response upon microbial infections. The humoral response relies in the massive production (up to 300 μM) of antimicrobial peptides (AMPs) mainly from fat bodies, a functional equivalent to the mammalian liver [85]. The cellular immune response of Drosophila involves the three subpopulation of circulating hemocytes: plasmatocytes, lamellocytes, and crystal cells. Plasmatocytes, the most abundant circulating hemocytes, are equivalent to mammalian macrophages, acting as professional phagocytes and releasing a large range of cytokines and AMPs. Interestingly, accumulating experimental evidence suggests a potential alternative role of AMPs as signaling molecules regulating different biological function in various tissues independently of their classical antimicrobial function [86]. For instance, as in humans, in Drosophila the AMPs tend to gradually and significantly increase during the aging [46,48,50,51] and, importantly, overexpression of certain AMPs (including Attacin-A) may lead to cognitive decline, neurotoxicity, and a decrease in lifespan [87,88].

We have previously demonstrated that ubiquitous expression of LRRK2 R1441C (by actin-GAL4 driver) leads to a toxic phenotype in Drosophila [48] that, at least in our case, was not observed by pan-neuronal expression (either by elav- or nSyb-GAL4 drivers). LRRK2 R1441C expressing flies show a strong inflammatory status in older animals monitored by inflammatory peptide (AMPs) and, in particular, two AMPs (Attacin-A and Drosocin) are already over-expressed at 7 days after eclosion, when no motor defects or neurodegeneration signs are detected in transgenic flies [35].

**Figure 2:**
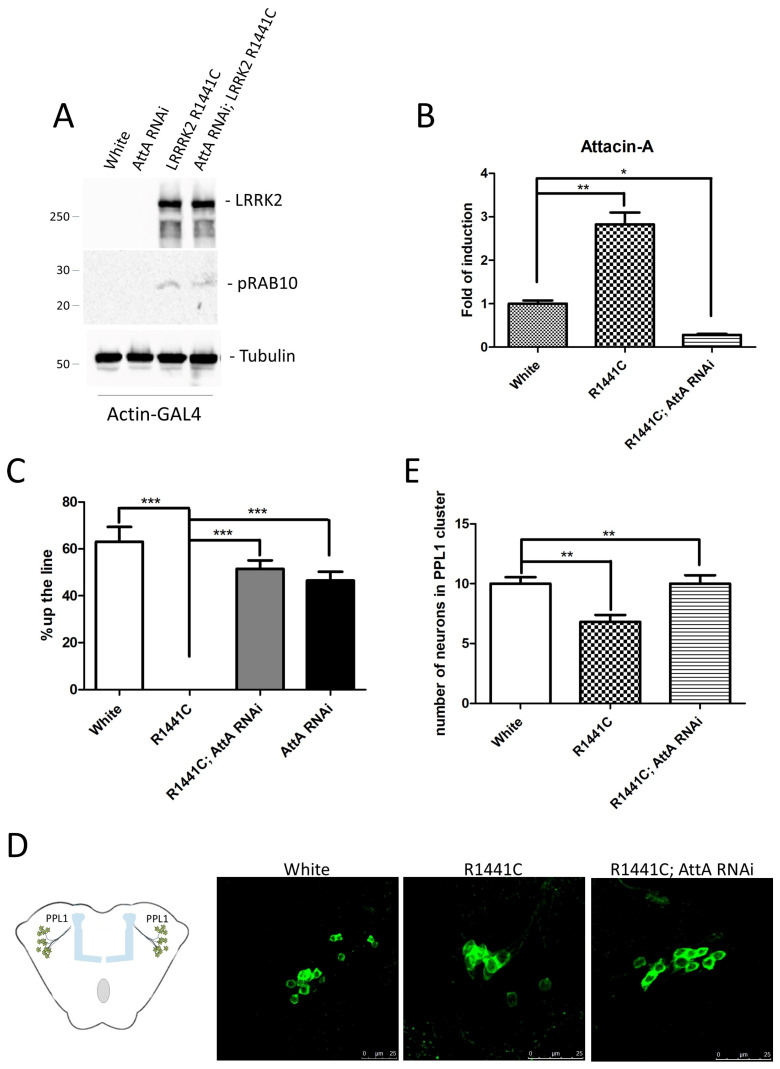
Analysis of AttA RNAi expression on the toxic phenotype due to LRRK2 R1441C expression under the control of actin-GAL4 driver. Transgenic lines used: Actin-GAL4-White (White), Actin-GAL4-UAS-LRRK2 R1441C (R1441C), Actin-GAL4-UAS AttA RNAi (AttA RNAi), Actin-GAL4-UAS-LRRK2 R1441C; UAS-AttA RNAi (R1441C; AttA RNAi). (**A**) Evaluation of LRRK2 and phospho-RAB10 expression by western blot analysis in head protein extracts of the indicated genotypes in 7-day-old animals. Anti-Tubulin antibody was used to normalize the different protein samples. (**B**) Evaluation of AttA expression by Real-Time PCR using RNA extracted from the heads of the different genotypes in 7-day-old animals. L32 was used as control to normalize the different samples. * *p* < 0.05 and ** *p* < 0.005. (**C**) Evaluation by climbing assay of locomotor activity of the different 45-day-old Drosophila lines. *** *p* < 0.001. (**D**) Schematic representation of PPL1 cluster localization in Drosophila brain and staining of dopaminergic neurons in PPL1 cluster of 45-day-old Drosophila lines of the different genotypes by immunofluorescence on whole brains using anti-TH antibody. (**E**) Quantification of dopaminergic neuronal number in the PPL1 cluster of the different genotypes. ** *p* < 0.005.

In an attempt to evaluate the inflammatory contribution of AttA inflammatory peptide in LRRK2 neuronal toxicity, we generated flies having both UAS-LRRK2 R1441C and an UAS-AttA RNAi. The flies carrying the double transgenes and flies carrying the single transgene as controls were crossed with the actin-GAL4 drivers. One week after eclosion, the LRRK2 expression and RAB10 phosphorylation were analyzed in the different lines by western blot. As illustrated in Figure 2A, the presence of two transgenes does not alter either LRRK2 expression or the increase in RAB10 phosphorylation due to LRRK2 R1441C expression. As expected, the presence of AttA RNAi decrease the AttA expression in the transgenic flies (Figure 2B) analyzed by Real-Time PCR. At 45 days after eclosion, the different flies were analyzed for locomotor activity by climbing assay. The concomitant expression of AttA RNAi is able to significantly rescue the climbing defects due to LRRK2 R1441C expression (Figure 2C). Finally, in 45-day-old flies, dopaminergic neurons in the Protocerebral Posterior Lateral 1 (PPL1) cluster were analyzed by immunofluorescence, using an antibody against Tyrosine Hydroxylase (TH) (Figure 2D,E). Of course, although the single UAS-AttA RNAi line has been characterized so far, we may not exclude the possibility that the RNA interference may affect other unrelated cellular pathways.

## 5. Discussion

In studies on Parkinson’s disease, Drosophila LRRK2 models largely confirm, validate, or sometimes anticipate, the findings arising from higher eukaryote experimental models. Expression of human or Drosophila LRRK2 variants in Drosophila has allowed the direct observation of the effects of mutations on behavior, neurodegeneration, and alterations in various cellular processes. Interestingly, flies expressing mutant forms of LRRK2 often show reduced lifespan, altered movement, and neuronal degeneration similar to that observed in patients with Parkinson’s disease. A particular advantage of using Drosophila as an experimental model is the availability of a large number of genetic tools both in terms of GAL4 drivers (to express the gene of interest in specific cell types) and in terms of fly lines that over- or hypo-express specific genes (to analyze the crosstalk between different signaling pathways).

Recent studies, in human and experimental models, have shown that LRRK2 is expressed at higher levels in immunocompetent cells compared to other cell types, including neurons, making it a possible key regulator of immune functions. Indeed, LRRK2 is strongly expressed in cells of the innate immune system such as neutrophils, macrophages, monocytes, and in the brain in astrocytes and microglia cells [84], both of which are considered part of the innate immune system based on the production of specific immune modulators and the expression of specific cell membrane receptors [89]. Interestingly, in the brain cells, there is a consistent convergence that LRRK2 exerts a pro-inflammatory effect, whereas in systemic immune cells the LRRK2 role on inflammatory cytokines/chemokines generation is still under debate [90].

Several reports underline a significant increase in neuroinflammation in PD patients. For example, post-mortem analysis of affected brain regions of PD patients shows activated complement components and reactive microglia [28]. Accordingly, different studies have reported elevated levels of inflammatory cytokines such as TNF, IL-1b, and IL-6 in both serum and CSF [91]. In addition, higher levels of inflammatory biomarkers appear to correlate with worse motor function and cognitive impairment in PD patients [92]. Interestingly, evidence from human and animal experimental models suggests that inflammation, due to an initial stimulus, may persist for many years and that chronic inflammation may be detrimental to neurons. All these findings point to chronic inflammation as a possible cause or contributing factor in the development of PD. In animal models, a number of anti-inflammatory compounds offer neuroprotection, while in PD patients, although epidemiological and preclinical studies suggest a possible beneficial effect, clinical trials have failed to provide robust evidence of their efficacy in the symptomatic phase of the disease [93]. It may be worthwhile to evaluate the anti-inflammatory effect in the pre-symptomatic phase to inhibit the initial pathological mechanisms before the likely irreversible neuronal decline in the symptomatic phase.

Several lines of evidence from human samples and animal experimental models suggest that pathogenic PD signs (e.g., alpha-synuclein misfolding) begin in the gastrointestinal tract and subsequently spread to vulnerable brain regions [94]. Experimental models suggest that the interplay between the gut microbiome or exogenous toxicants and intestinal immune system, may act as triggers or modifiers of neuronal pathology, further supporting the contribution of immunity, and in particular, the innate immune system to PD onset or progression [94]. LRRK2 is highly expressed in the innate immune cells and significantly upregulated by inflammatory modulators such as IFN-γ [95,96], a central cytokine in the control of the immune response. In turn, LRRK2 enhances the NF-κB-dependent transcription of inflammatory molecules in response to various stimuli [95,96], strongly suggesting a possible potential role in the communication between gut, immune, and nervous system [84].

**Figure 3:**
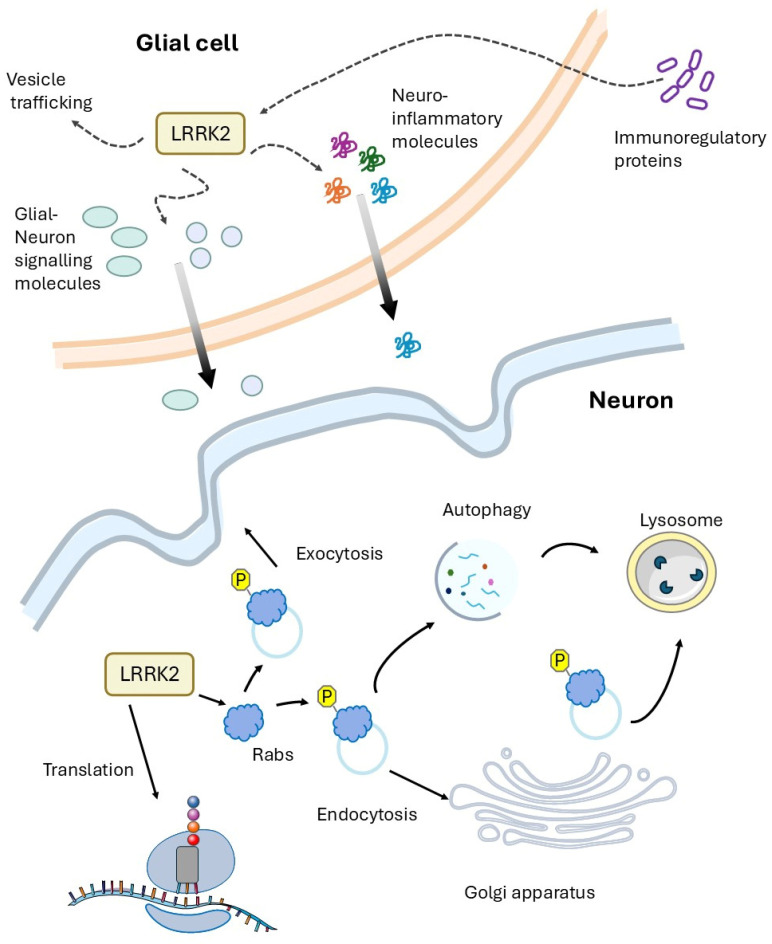
LRRK2 is highly expressed in glial cells compared to neurons. Several experimental results, in neurons, indicate an important role for LRRK2 in vesicle trafficking, autophagy, lysosome function, and protein translation. In glial cells, in addition to alterations in these molecular pathways, an alteration in the production of inflammatory molecules or molecules/proteins involved in neuronal-glial cell communication has been demonstrated. In addition, LRRK2 is significantly induced by immunoregulatory proteins such as by interferon-γ, further supporting the role of LRRK2 at the interface between neuronal and non-neuronal cells.

In Drosophila, we have detected a strong increase in inflammatory markers when LRRK2 pathological mutants are expressed either in all tissues (via actin-GAL4 driver) or in glial cells (via repo-GAL4 driver). Interestingly, here we show that the reduction in the expression of AttA inflammatory peptides by RNAi significantly rescues the LRRK2 R1441C phenotype in terms of motor performance and neurodegeneration. Figure 3 shows the potential role of LRRK2 in neuronal and glial cells, as suggested by experimental evidence from the Drosophila and higher eukaryotic models previously described. Taken together, these experimental results suggest that a non-cell-autonomous mechanism may contribute to PD LRRK2-related disease.

In conclusion, LRRK2 is an important gene in the context of neurodegenerative diseases, and non-rodents provide a highly useful model for exploring the pathogenetic mechanisms associated with this gene. The combination of non-rodent genetics and molecular biology may significantly contribute to the understanding of the role of LRRK2 in Parkinson’s disease and pave the way for new therapeutic possibilities, including compounds capable of reducing neuroinflammation. Up to the present, a general approach of anti-inflammatory therapy, although producing promising findings in pre-clinical experimental models, failed to translate into a clinical therapy. Likely, the identification of some certain inflammatory actors that play a key role in gut-immune-nerve communication could become specific therapeutic targets with a greater chance of success, especially if used in the pre-symptomatic phase of the disease.

## 6. Materials and Methods

### 6.1. Drosophila Lines

All fly stocks were maintained in our laboratory on standard cornmeal medium at 25 °C on a 12 h:12 h light:dark cycle. The Actin-GAL4 driver (BDSC #4414) and UAS-AttA RNAi (BDSC #56904) were from Bloomington Stock Center (Bloomington, IN, USA). The UAS-LRRK2-R1441C was a generous gift from prof. Cheng-Ting Chien (National Taiwan University Hospital Yun-Lin Branch, Taipei, Taiwan).

### 6.2. Evaluation of mRNA Expression by RT-PCR

Total RNA was extracted from Drosophila heads (5 male and 5 female) for each genotype in 500 µL of TRI-ZOL solution according to the manufacturer’s instruction (Life Technology, Thermo Fischer Scientific, Waltham, MA, USA). The RNA (0.5 µg) was converted to cDNA by AMV reverse transcriptase (Promega, Madison, WI, USA) at 37° for 1 h. Diluted (1:10) cDNAs were amplified by PCR (94° 30 s, 55° 30 s, 72° 30 s) in the presence of SYBR green (Biorad, Hercules, CA, USA) using the primers indicated in [97] (for RpL32 GACGCTTCAAGGGACAGTATCTG and AAACGCGGTTCTGCATGAG; for AttA CCCGGAGTGAAGGATG and GTTGCTGTGCGTCAAG) by Rotor-Gene Q Thermocycler. The delta-delta Ct method (2^−∆∆Ct^) normalized by machine internal software was used to evaluate the RNA content between the different genotypes.

### 6.3. Western Blot Analysis

Western blot analysis was performed as previously described [51]. Briefly, total body protein extracts were prepared by direct lysis in Laemmli buffer, resolved by standard sodium dodecyl sulphate-polyacrylamide gel electrophoresis, and subsequently transferred into nitrocellulose membrane (Life Technology). The membranes were incubated with 3% low-fat milk in 1X PBS-Tween 0.05% solution with the following antibody: anti-LRRK2 (1:5000 MJFF2 c41-2 Epitomics, Burlingame, CA, USA), anti-phospo-RAB10 (T73) (1:1000 MJF-R21 Abcam, Cambridge, UK), anti-alpha-tubulin (1:1000 12G10 DSHB, Iowa City, IA, USA), for 16 h at 4 °C. Goat anti-mouse immunoglobulin G (IgG) peroxidase-conjugated antibody (1:2500 Millipore Corporation, Burlington, MA, USA) or goat anti-rabbit IgG peroxidase-conjugated antibody (1:5000 Millipore Corporation) were used to identify immunocomplexes by enhanced chemiluminescence (Millipore Corporation).

### 6.4. Whole-Mount Immunostaining of the Adult Drosophila Brains

Fluorescent immunostaining was performed as previously described [51]. Briefly, whole adult brains were dissected. Cohorts of 6 to 10 flies per genotype were used each time. Brains were fixed for 20 min using 4% paraformaldehyde in phosphate-buffered saline (PBS), pH 7.4. After fixation, brains were permeabilized with 0.3% Triton X-100 in PBS and incubated in blocking buffer (5% normal goat serum in PBS 1X-0.3% Triton X-100) for 1 h at RT. Subsequently, primary anti-body anti-TH (1:500 AB152, Millipore) diluted in blocking buffer was incubated for 48 h at 4 °C. After extensive washing, the brains were incubated by secondary antibody Alexa Fluor^®^ 546 (Life Technology) diluted 1:1000 in blocking solution for 48 h at 4 °C. Finally, the brains were mounted using Mowiol mounting medium and fluorescence was revealed with a Leica TCS SP5 confocal microscope with LAS lite 170 image software (LAS AF 2019). DA neurons located in the PPL1 were counted in both hemispheres after Z-stack image acquisition to visualize all TH-positive neurons. A minimum of 10 brains were analyzed for each genotype in every experiment. Three independent experiments have been performed. Statistical analysis was performed with One-way ANOVA followed by a Bonferroni post hoc test.

### 6.5. Climbing Assay

Flies were age and sex matched, randomly selected, anesthetized with ice, and placed in conical tubes with a diameter of 2 cm. After 15 min of recovery at 25 °C, the flies were tapped to the bottom of the tube, and their subsequent climbing activity was quantified as the percentage of flies that reached 5 cm in 10 s. Any experimental sample was performed in duplicate (each with 15 flies) and the assay was repeated three or four times.

### 6.6. Statistical Analysis

The results are presented as means ± SEM of independent experiments as indicated. Statistical evaluation was conducted by One-way ANOVA and Bonferroni’s multiple comparison post-test. Values significantly different from the relative control are indicated with one, two, or three asterisks when *p* < 0.05, *p* < 0.005, and *p* < 0.001, respectively.

## Figures and Tables

**Table 1:** LRRK2 Drosophila models.

Genetic Modification	Expression	Phenotype	Refs.
dLRRK loss-of-function: LRRK^ex1^, generated by imprecise excision of the EP-element in the G7459 line	No expression	Severely impaired locomotor activity at 3 days after eclosion. Reduced TH-positive neurons	[40]
dLRRK loss-of-function P-element insertion line	No expression	Viable; reduced female fertility; increased DA content; more resistant to oxidative stress (paraquat/H_2_O_2_). No significant change in TH-positive neurons	[41]
Truncated dLRRK: P-element insertion in dLRRK gene causing the C-terminal kinase deletion	Endogenous truncated dLRR	More sensitive to H_2_O_2_ stress No sensitivity to paraquat, rotenone, beta-mercaptoethanol. No significant change in TH-positive neurons	[39]
Transgenic expression in dopaminergic neurons: UAS-dLRRK WT or Y1383C or I1915T/TH-GAL4 or Ddc-GAL4	DA neurons	Selective loss of dopaminergic neurons Locomotor dysfunction Early mortality	[41]
Transgenic expression UAS-LRRK2/TH-GAL4 or Ddc-GAL4	DA neurons	Selective loss of dopaminergic neurons Locomotor dysfunction Early mortality	[43]
Transgenic expression UAS-LRRK2-G2019S/TH-GAL4 or Ddc-GAL4	DA neurons	Loss of TH-positive neurons Locomotor dysfunction (improved with L-DOPA)	[43,44,45,46]
Transgenic expression UAS-LRRK2-G2019S/Ddc-GAL4	DA neurons	Loss of TH-positive neurons Neuron-glial BMP-signaling cascade is critical for mediating age-dependent neurodegeneration.	[47]
Transgenic expression UAS-LRRK2 WT or mutant/elav-GAL4	Pan-neuronal	Less severe or absent phenotype compared to DA neuron expression	[43,48]
RNA interference UAS-dLRRK-RNAi/repo-GAL4	Pan-glial	Locomotor deficits Glial apoptosis DA neurodegeneration	[49]
Transgenic expression UAS-LRRK R1441C/repo-GAL4	Pan-glial	Locomotor deficits DA neurodegeneration	[48]
Transgenic expression UAS-LRRK2-G2019S/Actin-GAL4	Ubiquitous	Loss of TH-positive neurons Locomotor deficits Increased inflammatory signals	[48]

## Data Availability

The data underlying the findings presented in this study are available upon reasonable request.
